# Malaria impact of large dams in sub-Saharan Africa: maps, estimates and predictions

**DOI:** 10.1186/s12936-015-0873-2

**Published:** 2015-09-04

**Authors:** Solomon Kibret, Jonathan Lautze, Matthew McCartney, G. Glenn Wilson, Luxon Nhamo

**Affiliations:** Ecosystem Management, School of Environmental and Rural Science, University of New England, Armidale, NSW 2351 Australia; International Water Management Institute, Pretoria, South Africa; International Water Management Institute, Vientiane, Laos

**Keywords:** Malaria, Dam, Reservoir-shoreline, Stable, Unstable, Sub-Saharan Africa

## Abstract

**Background:**

While there is growing recognition of the malaria impacts of large dams in sub-Saharan Africa, the cumulative malaria impact of reservoirs associated with current and future dam developments has not been quantified. The objective of this study was to estimate the current and predict the future impact of large dams on malaria in different eco-epidemiological settings across sub-Saharan Africa.

**Methods:**

The locations of 1268 existing and 78 planned large dams in sub-Saharan Africa were mapped against the malaria stability index (stable, unstable and no malaria). The *Plasmodium falciparum* infection rate (PfIR) was determined for populations at different distances (<1, 1–2, 2–5, 5–9 km) from the associated reservoirs using the Malaria Atlas Project (MAP) and WorldPop databases. Results derived from MAP were verified by comparison with the results of detailed epidemiological studies conducted at 11 dams.

**Results:**

Of the 1268 existing dams, 723 are located in malarious areas. Currently, about 15 million people live in close proximity (<5 km) to the reservoirs associated with these dams. A total of 1.1 million malaria cases annually are associated with them: 919,000 cases due to the presence of 416 dams in areas of unstable transmission and 204,000 cases due to the presence of 307 dams in areas of stable transmission. Of the 78 planned dams, 60 will be located in malarious areas and these will create an additional 56,000 cases annually. The variation in annual PfIR in communities as a function of distance from reservoirs was statistically significant in areas of unstable transmission but not in areas of stable transmission.

**Conclusion:**

In sub-Saharan Africa, dams contribute significantly to malaria risk particularly in areas of unstable transmission. Additional malaria control measures are thus required to reduce the impact of dams on malaria.

## Background

Construction of large dams—water infrastructure with a crest height greater than 15 m, or a storage capacity exceeding 3 million cu m for heights between 5 and 15 m [1]—has been widely recognized as a key factor in promoting economic growth, ensuring food security, alleviating poverty, and increasing resilience in the face of climate variability and change in sub-Saharan Africa (SSA) [2–4]. The World Bank has applied language such as ‘infrastructure gap’ to the paucity of the continent’s dams and water storage capacity [2, 5], and the African Ministers Council on Water declared that Africa is “held hostage” by its hydrology due to the deficit of water infrastructure [6]. Encouraged by the increased volume of international aid for water resource development, SSA has, in recent years, experienced a new era of large dam construction to address pressing challenges related to food security and increasing demands for economic development [7].

Environmental modification such as dam construction has long been recognized to enhance malaria transmission, a disease that globally claims an estimated 627,000 lives each year, 90 % of which are in SSA [8]. In Africa, increased malaria incidence following dam construction has been reported around the Bamendjin Dam in Cameroon [9], the Kamburu Dam in Kenya [10], the Koka reservoir in central Ethiopia [11, 12], the Gilgel Gibe Dam in southwest Ethiopia [13], the Manyuchi Dam in Zimbabwe [14], and the Akosombo Dam in Ghana [15].

While a cursory review may lead to the presumption that dams’ impacts on malaria are important and negative, there is evidence that the dynamics of malaria transmission around reservoirs may be more complicated. For example, no increase in malaria was reported around the Manantali Dam in Mali [16] and the Foum Glaita Dam of Mauritania [17]. Lack of a malaria impact around these two dams appears mainly due to the replacement of the most efficient vector (*Anopheles gambiae* sensu stricto and *Anopheles funestus*) by less anthropophilic species (*Anopheles arabiensis* and *Anopheles pharoensis*) that use the shoreline environment as breeding habitat [16, 17].

Despite the growing evidence pointing to a potentially major cumulative effect of dams on the malaria burden of SSA, and the important nuance on variation in dam impacts on malaria in diverse eco-epidemiological settings, neither issue has been systematically investigated. Keiser et al. [18] reviewed the literature and found that 3.1 million people are at risk of malaria due to large dams in SSA, but stopped short of determining the aggregated contribution of dams to malaria burden across the region. Another study [19] examined how the effects of environmental management could offset malaria transmission in different eco-epidemiological settings; these authors nonetheless did not rigorously explore how malaria impacts differ between alternative eco-epidemiological contexts.

The present study investigated the impact of current and planned large dams on malaria across SSA. Dams with georeferenced locations were mapped in relation to areas of stable and unstable malaria transmission. The population at risk of malaria in different epidemiological settings was estimated and the difference in malaria infection rate at different distances from the reservoirs analysed. Finally, the contribution of these dams to the malaria burden in the region was determined.

## Methods

### Study area

**Fig. 1:**
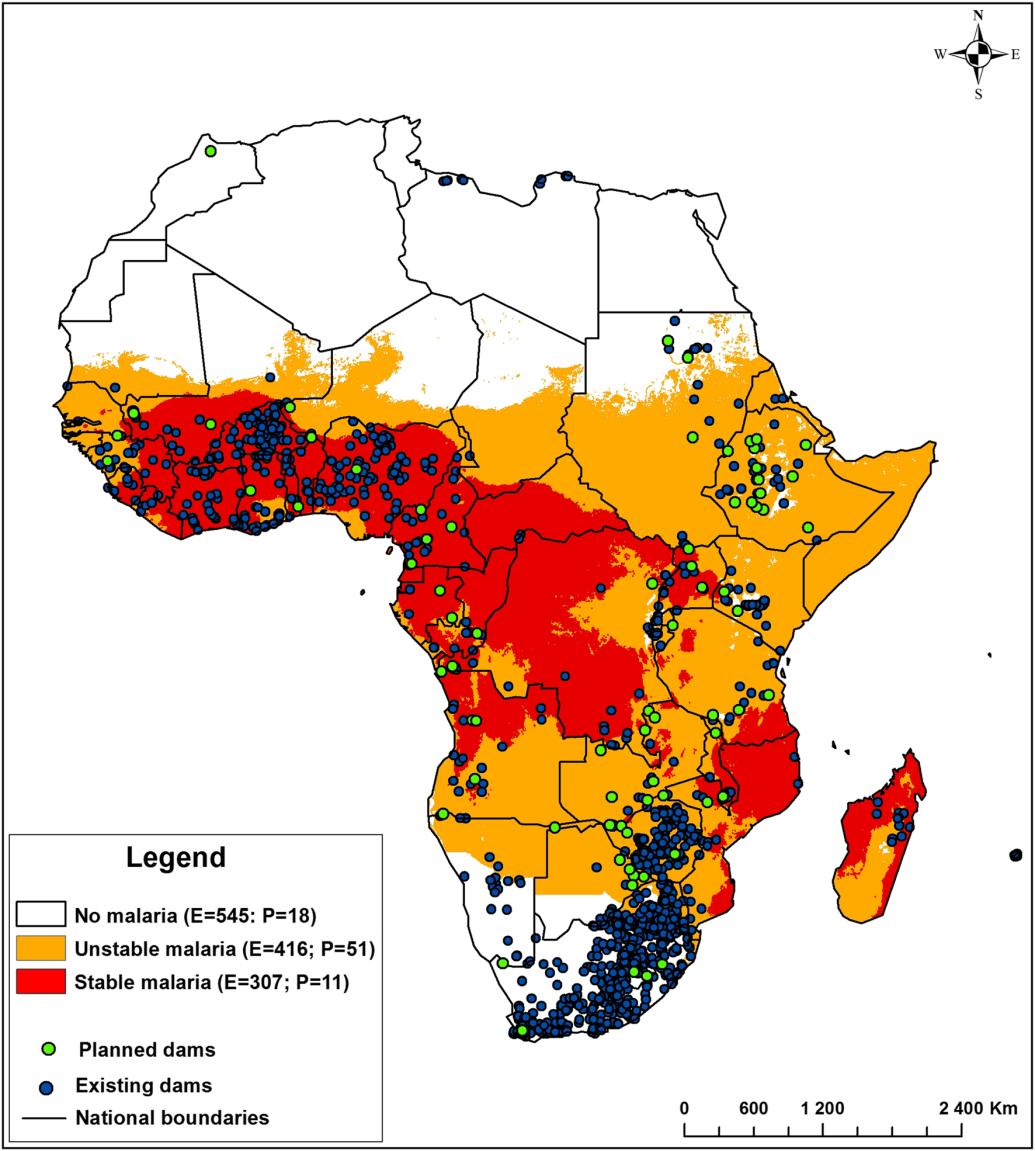
Map showing spatial distribution of existing and planned dams in Africa with respect to the 2010 malaria stability indexing (*E* no. existing dams, *P* no. planned dams) (adapted from Kibret et al. [22])

This study focused on SSA—geographically, the area of the African continent that lies south of the Sahara Desert. It consists of all African countries except Morocco, Algeria, Tunisia, Libya, and Egypt [20]. This region accounts for the greatest burden of malaria in the world where *Plasmodium falciparum*, the most severe of the malaria parasite species that infect humans, is predominant. Annually, an estimated 174 million cases occur in this region [8]. Malaria transmission is generally stable in western and central Africa, unstable in much of eastern Africa and unstable to absent in southern Africa [21] (Fig. 1).

### Data collection

The present study used available databases to quantify the impact of dams on malaria. Dam databases were used to collect information on the number and location of dams across SSA. Population data and malaria prevalence databases were used to estimate population at risk around dams in different eco-epidemiological settings of SSA.

### Data on existing and planned African dams

To identify and locate existing and planned dams in SSA, georeferenced locations of individual dams (both existing and planned) were obtained from the FAO African dams database [23] and the International Rivers database [24]. Data on water storage capacity, dam height and reservoir surface area were obtained from the ICOLD World Register of Dams [25] and the Global Reservoirs and Dams (GRanD) database [26]. Locations and parameters of additional dams were obtained from a number of journal articles, project reports and dissertations.

Overall, georeferenced locations and dam parameters were gathered for a total of 1268 existing dams (out of an estimated total of over 2000 [7]) and 78 planned dams (out of an estimated total of 150 [24]) in SSA. A planned dam was defined as a dam currently under construction or planned for construction in the next 5 years. While the number of existing and planned dams for which locations could be found was below the known total of each, the set of existing and planned dams mapped for this study is the most extensive yet utilized in an analysis of the malaria impacts of dams in SSA.

### Estimating reservoir perimeters

Data on reservoir perimeter are necessary to estimate the population at risk of malaria due to a dam. However, these data are not easily available for most dams. Thus reservoir perimeter was estimated using a method proposed by Lehner et al. [27] and Keiser et al. [18]. First, it was assumed that reservoirs have a rectangular shape [18]. Length of reservoir (LR) was calculated for each dam according to LR = A/LD, where A represents the surface area of the reservoir and LD the length of the dam. A and LD were obtained from the World Register of Dams and FAO database, respectively. Then, the perimeter of the reservoir was estimated as 2LR + 2LD. For each dam, the calculation was based on the reservoirs’ maximum water storage (i.e., the reservoir at full supply level, when the surface area is at a maximum).

The ‘rectangular’ reservoir shape assumption was validated using known reservoir perimeters from the literature. The actual shape of 11 reservoirs, derived from the literature review, indicated that dams with reservoir size >1000 sq km had a much longer reservoir length than dam length (median LR/LD = 65.4) while dams with reservoir size <100 sq km had comparable dam and reservoir lengths (median LR/LD = 4.2). This supports the ‘rectangular’ reservoir shape approach in the present study, which assumed a much greater length than width.

It is also recognized that in many cases the reservoir water level varies substantially throughout the year and this will change both the perimeter of the reservoir shoreline and the relative distance of the shoreline to communities. However, data on fluctuations in reservoir water levels are not generally available, so it was not possible to make allowance for any temporal variability in reservoir surface area.

### Data on malaria transmission stability

The Gething et al. [21] classification was utilized to characterize the epidemiological settings in which the dams were located. This is defined as:

- Stable transmission in areas with annual *P. falciparum* infection rates (PfIR) greater than 0.1 cases per 1000 population;
- Unstable transmission in areas with annual PfIR between 0 and 0.1 cases per 1000 population;
- No malaria in areas having zero annual PfIR.

### Data on malaria transmission

The Malaria Atlas Project (MAP) database was used to produce annual predictions of spatial PfIR rates at high resolution (1 × 1 km grid) [21]. MAP is an initiative founded in 2005 to generate new and innovative methods of mapping malaria risk and has continuously updated georeferenced PfIR surveys since 2005. The updated version, completed on 1 June 2010, consisted of 22,212 quality-checked and spatiotemporally unique malaria prevalence survey data points. The 2010 dataset was used to determine annual PfIRs for populations at different distances from reservoirs in areas of stable and unstable transmission [21]. All dams classified as ‘existing’ in this study were commissioned before 2010. Additional data were obtained from literature review and the World Health Organization [28].

### Literature review

A systematic review of the peer-reviewed literature, dissertations and technical reports was carried out, with an emphasis on published research findings from assessments of the impact of large dams on malaria transmission. Articles were searched mostly through PubMed using the combination of keywords such as ‘malaria’, ‘*Anopheles* vector’, ‘dams’, ‘mosquito breeding’, ‘reservoir shoreline’ and ‘sub-Saharan Africa’. Relevant references cited by each reviewed study were also examined. Pertinent book chapters and websites (e.g., 27) were also consulted. Two types of studies were included: (1) those that assessed epidemiological (malaria prevalence or incidence) and/or entomological (malaria mosquito bionomics, density and vectorial capacity) variables before and after the construction of a dam; and, (2) those that compared dam/reservoir villages and non-dam/reservoir settings with similar social and eco-epidemiological settings were included. Studies without a control comparison design were excluded from this review to ensure causality in the environmental factors responsible for changes in malaria transmission in nearby villages.

A total of 17 studies showing the effects of 11 large dams on malaria incidence and/or vector breeding in SSA were found. The impact of dams on malaria was analysed in relation to areas of stable and unstable transmission.

### Data analysis

#### Mapping dams and malaria

The distribution of existing and planned dams was overlaid on the malaria stability index map using ArcGIS and the number of large dams in each malaria stability category (stable, unstable and no malaria) was determined. ArcGIS was used to produce all the maps and for population estimates.

#### Estimating the population at risk around dams

To estimate the population at risk at different distances from a dam and its associated reservoir, high resolution (1 × 1 km) Worldpop Project population distribution database [29] was used. Population at risk was estimated as all persons living within a 5-km distance of the reservoir, upstream of a dam. The impact of a dam on malaria was assumed to be negligible beyond 5 km due to mosquitoes’ limited flight range [30].

#### Malaria incidence around dams

Using the MAP database, annual PfIR was computed for four distance cohorts (i.e. <1, 1–2, 2–5, 5–9 km). These cohorts lie within the same climatic region at each of the dam sites. The 5–9 km cohort was taken as the control group for each dam: the assumption being that malaria incidence in this zone equated to what would have occurred in the other distance cohorts if the dam had not been built. The Odds Ratio (OR) (i.e., the ratio of malaria in each cohort relative to the control) was calculated to compare the PfIR among the cohorts. The annual number of malaria cases for each cohort was calculated by multiplying PfIR by the population present in that cohort.

Since the population density varies among the cohorts, the difference between PfIRs between at risk cohorts (<1, 1–2 and 2–5 km) and the control cohort (5–9 km) was compared as follows [31]:

$$z = \frac{{(\hat{\rho }_{1} - \hat{\rho }_{2} ) - 0}}{{\sqrt {\hat{\rho }(1 - \hat{\rho })\left( {\frac{1}{{n_{1} }} + \frac{1}{{n_{2} }}} \right)} }}$$

where, $\hat{\rho }_{1}$ is the PfIR (in per cent) in the at risk cohort, $\hat{\rho }_{2}$ is the PfIR (in per cent) in the control cohort, $\hat{\rho }$ is the odds ratio of $\hat{\rho }_{2}$ and $\hat{\rho }_{1}$, and n_1_ and n_2_ are population size of at risk and control cohorts, respectively, *z* is a value on the *Z*-distribution.

#### Determining the increased cases associated with dams

The annual number of malaria cases associated with current and future dams was determined for unstable and stable transmission areas. The number of annual malaria cases attributable to dams was estimated by calculating the difference in the number of annual malaria cases for communities less than 5 km and for communities greater than 5 km (i.e., 5–9 km) from the reservoir, allowing for differences in population size. The annual PfIR calculated for 5–9 km was applied to the <5-km cohorts multiplied by the population in the <5-km cohorts. In the planned dams, the rate of malaria case increase between at risk (<5 km) and control (5–9 km) cohorts in the existing dams was taken to predict the potential increase in malaria cases in planned dams after dam construction—with differential rates for stable and unstable areas. No adjustment was made for the population growth that often accompanies dam construction.

#### Validating with evidence from literature

A total of 17 dam-malaria studies focused on 11 dams explored relationships between dams and malaria. For the 11 dams where literature is available, results from the MAP-based analysis and those reported in the literature were compared. Due to limitations in the results reported in the literature, it was not possible to determine malaria incidence for the four distance cohorts used in the MAP-based analyses. However, the data were sufficient to enable the range of malaria prevalence and OR to be calculated for those living close (<3 km) to dams and further away (>3 km) in stable and unstable areas. For the MAP data, two distance groups were recreated [<3 and >3 km (3–6 km)] to enable comparison with the literature dataset. The OR of malaria prevalence from MAP and literature were compared using the Chi square test. Statistical analyses were done using statistical software, SPSS version 22 (SPSS Inc, Chicago, IL, USA). The level of significance was determined at the 95 % confidence interval (*P* < 0.05).

## Results

### Spatial distribution of dams in Africa

Dams are distributed across stable, unstable and no malaria transmission areas of SSA (Fig. 1). Among the 1,268 existing dams, 33 % (n = 416), 24 % (n = 307) and 43 % (n = 545) are located in areas of unstable, stable and no malaria transmission, respectively. Dams in stable transmission areas are largely distributed across western Africa, while dams in unstable areas and no malaria areas are mainly located in southeast and southern Africa, respectively. Of the planned dams, 65.4 % (n = 51) are located in areas of unstable transmission, 11.5 % (n = 9) in areas of stable transmission, and 23.1 % (n = 18) in areas without malaria.

### Population at risk of malaria around dams

Approximately 20 million people in SSA (2 % of the total population) live within 5 km of the 1268 reservoirs investigated in this study (Table 1). Of these, 14.6 million (1.42 % of the total population of SSA) live in areas at risk of malaria: 6.4 million in areas of stable transmission and 8.2 million in areas of unstable transmission. In addition, approximately 442,000 people currently live within 5 km of the reservoirs associated with the 60 planned dams located in areas at risk of malaria transmission (i.e., either stable or unstable) (Table 2).

**Table 1:** Summary of number dams and people living around existing dams in stable, unstable and no malaria zones across sub-Saharan Africa

Malaria stability	Total population in the region		Total area (sq km)		No. dams (% from total)	No. people within 1 km radius from reservoirs	No. people living between 1 and 2 km from reservoirs	No. people living between 2 and 5 km from the reservoirs	Total population within 5 km radius from reservoirs
	Number	%	Area (sq km)	%					
Stable	298,682,666	30	7,086,268	30	307 (24.2)	310,216	1,128,271	4,967,740	6,406,226
Unstable	418,451,433	42	14,459,409	61	416 (32.8)	439,921	1,409,537	6,783,531	8,193,239
No malaria	273,355,662	28	2,042,223	9	545 (43.0)	1049	819,167	4,785,258	5,605,474
Total	990,489,781	100	23,587,900	100	1,268 (100)	751,186	3,356,975	16,536,529	20,204,939

**Table 2:** Summary of number dams and people living around planned dams in stable, unstable and no malaria zones across sub-Saharan Africa

Malaria stability	No. dams (% from total)	No. people within 1 km radius from reservoirs	No. people living between 1 and 2 km from reservoirs	No. people living between 2 and 5 km from the reservoirs	Total population within 5 km of the reservoirs
Stable	9 (11.5)	13,127	49,787	296,913	359,827
Unstable	51 (65.4)	3,610	13,291	65,085	81,986
No malaria	18 (23.1)	8,816	28,842	163,297	200,956
Total	78 (100)	25,553	91,920	525,295	642,769

### Malaria incidence around reservoirs derived from MAP

Malaria incidence in communities living closer to reservoirs was greater than those living farther away (Table 3). In areas of unstable transmission, annual PfIR was greater in communities living within 1, 1–2 and 2–5 km from reservoirs, than in those living 5–9 km away. This difference was statistically significant in the <1 km (*z* = −9.842; *P* < 0.05) and 1–2 km (*z* = −6.513; *P* < 0.05) cohorts. In areas of stable transmission, the annual PfIR in people living within 1, 1–2 and 2–5 km from reservoirs also appeared greater than for those located 5–9 km away. However, the differences in PfIR were not statistically significant among the cohorts (*X*^2^ = 6.252; df = 3; *P* > 0.05).

**Table 3:** Mean *Plasmodium falciparum* infection rate (PfIR) in communities in the vicinity of dams in stable and unstable areas of the sub-Saharan Africa

Malaria stability and cohort population	Mean PfIR	95 % CI	Odds ratio	*P* value
Stable				
<1km from reservoirs	35.18	23.04–47.32	1.92	>0.05
1–2 km from reservoirs	28.36	12.25–43.47	1.55	>0.05
2–5 km from reservoir	21.52	10.31–32.72	1.17	>0.05
5–9 km from reservoir (Control)	18.33	8.12–28.54	1	–
Unstable				
<1km from reservoirs	16.33	10.31–22.35	3.20	<0.05
1–2 km from reservoirs	11.31	8.22–14.40	2.21	<0.05
2–5 km from reservoir	9.54	6.21–12.87	1.87	>0.05
5–9 km from reservoir (Control)	5.11	3.03–7.19	1	–

### Annual number of malaria cases associated with large dams

In areas of unstable transmission, approximately 919,000 malaria cases per year were associated with the presence of the 416 dams. In areas of stable malaria transmission, 204,000 malaria cases per year were associated with the presence of the 307 dams (Table 4). Overall, allowing for differences in both the number of dams and population, the effect of dams on annual malaria cases was 3.5–4.5-fold higher in areas of unstable transmission than in areas of stable transmission. The data also suggest that malaria cases in areas of unstable malaria transmission were on average 3.2 times greater in communities living close to existing reservoirs than those living more than 5 km from them. Overall, the reservoirs investigated account for 0.6 % of the total malaria burden in the SSA. However, in the vicinity of the reservoirs in stable and unstable areas, on average reservoirs associated with large dams contribute to 47 % of malaria cases in communities living within 5 km of them.

**Table 4:** Estimates of annual malaria cases (using MAP database) attributable to proximity to reservoirs (<5 km) in stable and unstable areas of sub-Saharan Africa

	No. malaria cases <5 km	No. malaria cases <5 km (assuming similar case rate as >5 km)	No. malaria cases attributable to presence of dams
Existing dams			
Unstable	1,337,956	418,675	919,281
Stable	1,377,979	1,174,261	203,718
Planned dams			
Unstable	58,760	13,388	45,371
Stable	77,435	65,956	11,478

### Additional malaria cases associated with planned dams

Completion of the 78 planned dams assessed here is expected to exacerbate the local malaria burden—particularly in areas of unstable transmission. Making no allowance for possible population change, the 60 planned dams located in areas with malaria will add approximately 45,000 cases in areas of unstable transmission and about 11,000 cases in areas of stable transmission (Table 4).

### Malaria prevalence around dams derived from the literature

Previous studies generally confirm that large dams have a greater impact on malaria prevalence in areas of unstable transmission (Table 5). Fourteen studies around dams in areas of unstable malaria transmission indicated that malaria prevalence in villages located <3 km from the dams, was 2.3–19.9 times higher than in villages located >3 km from the dams. By comparison, the analyses of the MAP database for the same dam communities indicated a 3.6–24.5 greater malaria prevalence in the communities located close to reservoirs (Table 6). The differences in malaria prevalence (both derived from the literature and MAP) between those living close to (<3 km) and farther away (>3 km) from the reservoirs were statistically significant (Chi square test, *P* < 0.05). In stable areas, four studies indicated that malaria prevalence increased by 1.2–1.4 times in communities living within 3 km as compared to those living between 6 and 9 km from the reservoirs. By comparison, the analyses of the MAP database indicated a 1.1–1.8 fold increase in malaria prevalence in the communities located close to the reservoirs (Table 6). However, in neither case was the difference statistically significant in the stable areas. Overall, the estimated malaria prevalence using the MAP database was broadly consistent with that reported in the literature, increasing confidence that the results derived from the MAP analyses are reasonably valid.

**Table 5:** Documented malaria prevalence (from literature *vs* MAP database) around some African dams

Stable/unstable	Location	Documented impact (from literature)	Documented impact (MAP)	Source (reference)
Unstable Transmission	Manyuchi dam on Mwenzi river of Zimbabwe	0 % malaria before the dam, 28 % prevalence after the dam	22.4 % in <2 km, and 8 % in 5–9 km	[14]
	Bamendjin dam in Cameroon	36 and 25 % in communities living <3 km and >14 km, respectively	41 % in <2 km communities and 18 % in 5–9 km	[9]
	Kamburu dam on Upper Tana River Basine, Central Kenya	4.5 % in communities <4 km, and 1.8 % in those 11 km away	19 % in <2 km communities and 6.5 % in 5–9 km	[10]
	Masinga dam, eastern Kenya	26 % in communities <1 km and 7 % in those living >3 km from the dam	31 % in communities living <3 km and 11 % in those living within 5–9 km	[32]
	Gilgel-Gibe dam on Omo river of Ethiopia	PV^a^ prevalence was 7.7 % in communities within 3 km as compared to 4.4 % in those living >3 km from the dam.	MAP only documents Pf^b^ prevalence	[13, 33]
	Koka dam on Awash river of Ethiopia	9 % in communities <1 km; 0.53 in those 5–9 km away	25 % in <1 km and 4.5 % at 5–9 km	[11, 12, 34]
	Mai Nigus, Mai Sessela and Mai Seye dams in Highlands of Tigray, Northern Ethiopia	4.17 % in communities <3 km and 3.65 % in >6 km	2.1 % in < 2 km and 0.4 % in 5–9 km	[35, 36]
Stable transmission	Diama dam Senegal River Basin, Senegal	31 % before dam construction and 29 % after dam construction	39 % in <2 km and 24.5 % in 5–9 km	[37]
	Foum Glaita on Gorgor river, southeast Mauritania	26 % before construction and 21 % after construction	31 % within 2 km and 27 % within 5–9 km	[17]
	Akosombo dam, Ghana	24 % in 2004, 34 % in 2005 and 41 % in 2006—a 10 % increase annually in communities <3 km	39 % in 2010 in communities within 2 km	[15]
	Two dams in Middle Senegal River Basin, Northern Senegal	22 and 17 % in communities within 3 km and at 7 km, respectively	11 % in 2 km radius and 18 % in 5–9 km	[16]

Malaria prevalence was determined from MAP database based on the estimates of reservoir perimeter

*MAP* Malaria Atlas Project

^a^ *Plasmodium vivax*

^b^ *Plasmodium falciparum*

**Table 6:** Summary of comparison of malaria prevalence around some African dams using data from the literature and the MAP database

Malaria stability	Documented impact from literature (range)			Documented impact from MAP (range)		
	Malaria prevalence <3 km	Malaria prevalence >3 km	Odds ratio	Malaria prevalence <3 km	Malaria prevalence >3 km	Odds ratio
Unstable	4.2–36.1 %	1.5–25 %	2.3–19.9^a^	6.3–41.5	0.4–28 %	3.6–24.5^a^
Stable	22.0–32.3 %	17.3–29.2	1.2–1.4	28.8–52 %	25.6–62 %	1.1–1.8

*MAP* Malaria Atlas Project

^a^The malaria prevalence difference between dam and non-dam villages was significant (Chi square test, *P* < 0.05)

## Discussion

### The cumulative burden imposed by large dams is major

This study confirmed that dams generally intensify malaria transmission in SSA. The main findings are that the existing large dams investigated in this study increase the risk of malaria for close to 15 million people and contribute more than 1 million cases annually to the malaria burden of SSA. The planned dams investigated in this study will increase the risk for an additional 400,000 people and add more than 50,000 cases annually, based on current population densities. This number may increase significantly after the commissioning of these dams, as past experience [38] indicates that people tend to migrate towards the shores of reservoirs for livelihood purposes (mainly agriculture). The contribution of these dams to malaria burden in the region is thus substantial.

### Estimates of dam-associated malaria impacts are conservative

The present study included a large proportion of existing dams in the analyses. Nonetheless, there are believed to be at least 800 additional large dams in SSA for which georeferenced data were not available and so they were not included. Assuming that these 800 dams have the same approximate distribution in relation to areas of stable, unstable and no malaria as the 1268 dams that were mapped, a realistic estimate of the total number of cases attributable to existing large dams in SSA annually would be of the order of 1.8 million. It is believed that this estimate is conservative because a large proportion of the large dams (n = 502) for which georeferenced data are available are located in South Africa, where there is no malaria. This means that a greater proportion of the existing unmapped dams are located outside South Africa, most likely in areas of either stable or unstable transmission.

Similarly, a number of planned large dams were not included in the present study due to data limitations. The fact that approximately two-thirds of the planned dams are located in areas of unstable transmission should be concerning given their likely cumulative impacts in unstable areas. Incorporation of such dams into the analyses would undoubtedly render the figures provided above low estimates, as additional dams will further increase the malaria risk of dams in the region.

### Magnitude of dam-associated malaria in SSA has been underestimated

The present study showed that the population at risk of malaria around dams is at least four times greater than that previously estimated [18]. Reasons for the large difference in population at risk were mainly because the present study used a more robust dataset and a large number of dams for analysis. Comparison of the cumulative burden of malaria in SSA due to large dams with other studies is not possible, as attempts were not previously made to quantify dam-associated malaria cases. The present study is the first of its kind to quantify the impact of a high proportion of large dams on malaria in SSA.

### The impact of large dams is far more severe in areas of unstable transmission

The study confirmed previous assertions that the impacts of dams are much greater in areas of unstable transmission [18, 22]. A possible explanation for this is that malaria in stable areas is broadly continuous and water reservoirs simply add to a wide array of existing breeding habitat, available throughout the year. In contrast, in areas of unstable transmission, where malaria is seasonal, availability of mosquito breeding habitat in the dry season is one of the limiting factors for malaria transmission. Rainfall has been indicated as major determinant that limits the length of malaria transmission to the wet season in semi-arid areas [39]. In such cases, reservoirs may effectively create conditions suitable for mosquito development as they increase humidity throughout the year, thereby increasing vector abundance at times of year when they would not normally be found. Furthermore, the present study indicated that the impact of dams on malaria in unstable areas could either lead to intensified malaria transmission or change the nature of transmission from seasonal to perennial. Further study is needed to better understand the ecological and entomologic factors that lead to enhanced transmission in unstable areas.

### How important are large dams’ impacts on malaria among other anthropogenic changes?

This study indicated that there are at least 1.1 million malaria cases associated with current dams and most likely at least an additional 56,000 cases associated with future dams, each year in SSA. Throughout SSA, other environmental modifications such as irrigation [40], deforestation [41], small dams [42], and urbanization [43] have been identified as major anthropogenic determinants of malaria but the relative importance of these factors is unknown. More rigorous identification of the relative contribution of anthropogenic determinants to the total malaria burden could strengthen the allocation of resources to fight the disease.

### Dam-associated malaria could challenge SSA’s current struggle towards malaria elimination

Whilst Africa is currently recording success stories in reducing malaria and even considering malaria elimination [44], extensive dam construction could confound malaria control efforts. Indeed, despite growing evidence of the impact of dams on malaria, there is scant evidence of their negative impacts being fully offset. The only documented example that showed how to systematically control malaria around a large dam comes from the Tennessee Valley Authority in USA over 60 years ago [45]. Ultimately, the development and implementation of various conventional and unconventional approaches to mitigate malaria around large dams may be required in SSA. In particular, four unconventional approaches may be worth exploring (Lautze et al., unpublished): (1) dam placement—decision-making related to placement of dams in a river basin; (2) dam design and reservoir sizing—the degree of its operational flexibility and the nature and size of the reservoir; (3) reservoir operation and habitat modification—the way in which the dam is operated to allow measures to suppress larval development; and, (4) other environmental controls, such as introducing larvivorous fish. Furthermore, public health measures as part of dam planning (e.g., distribution of bed nets, blanket-treating all new migrants, construction of mosquito-proof houses, and improving local health facilities) should also be coordinated with existing malaria programmes.

### Climate change may exacerbate the impact of dams on malaria in SSA

Large dams have been promoted as a mechanism to adapt to the likely increase in hydrological variability arising from climate change in Africa [46]. However, changes in climate characteristics, in particular local temperature and rainfall may also affect malaria transmission [47]. Extended dry seasons during El Niño years have been associated with malaria epidemics in the highlands of East Africa [48]. Furthermore, increased temperatures can also increase the rate of blood feeding by female mosquitoes which results in intensified malaria transmission [49]. Recent studies also indicated that climate change will likely push the altitude limits of unstable malaria towards the highlands of East Africa [50, 51]. In contrast, in the lowlands where temperature is generally high, transmission decreases dramatically with increasing temperature above 28 °C [52]. Future studies are needed to investigate the possible impact of climate change on malaria around both existing and planned dams in SSA, including in areas that are currently malaria free.

### Limitations of this study

The major caveat of this study is that a number of environmental factors such as climate, land use and other seasonal and ecological drivers were not investigated. It was assumed that these potentially confounding factors affect equally both study groups—communities located within 5 km of a reservoir and those located more than 5 km from a reservoir. Approaches adopted in this study were consistent with those used in related literature (e.g., Keiser et al. [18]). In particular, in common with most similar studies, decoupling impacts of the river from the reservoir was not achieved. Given that mosquitoes typically breed in standing rather than flowing water, it was assumed that water reservoirs were more important contributors to malaria than the rivers flowing into and out of them. To undertake analysis that removes this limitation requires studies that evaluate the malaria situation before and after dam construction. To date, only one study is available that compares the malaria situation before and after dam construction [37].

In addition, the assumption that reservoir shapes are rectangular is an oversimplification. In the present study, limited verification was achieved using data from the literature. Nonetheless, in future research the analyses of the present study should be repeated using the actual reservoir shapes derived from high-resolution satellite data.

### Avenues for future investigation

Dams may enhance transmission in the main periods of transmission, or change the seasonal pattern of malaria transmission. This study was focused on establishing aggregate annual impacts of dams in areas of stable and unstable transmission. Future investigations can focus on seasonality of such malaria impacts, particularly in unstable areas, to enhance implementation of disease control efforts. Future investigations should also determine the nature of adverse impact on malaria as dams either intensify transmission or may change transmission from seasonal to perennial. Such studies will then help understand underlying factors that explain why certain dams have produced significant impacts, whilst others have produced only negligible impacts.

### The time is ripe for action

Current investment in large dams in SSA is increasing to respond to the need for urgent economic development. Results of the present study call for intensive measures to mitigate malaria in the vicinity of the reservoirs created by existing and planned large dams. Whilst recognizing the importance of dams for economic development, it is unethical that people living close to them pay the price of that development through increased suffering and, in extreme cases, loss of life due to disease. Those building dams must invest effectively in measures to prevent malaria transmission.
